# Shape recognition: convexities, concavities and things in between

**DOI:** 10.1038/srep17142

**Published:** 2015-11-24

**Authors:** Gunnar Schmidtmann, Ben J. Jennings, Frederick A. A. Kingdom

**Affiliations:** 1 McGill Vision Research, Department of Ophthalmology, McGill University Montreal General Hospital, L11, 416 1650 Avenue Cedar, Montréal, Québec, H3G 1A4 Canada

## Abstract

Visual objects are effortlessly recognized from their outlines, largely irrespective of viewpoint. Previous studies have drawn different conclusions regarding the importance to shape recognition of specific shape features such as convexities and concavities. However, most studies employed familiar objects, or shapes without curves, and did not measure shape recognition across changes in scale and position. We present a novel set of random shapes with well-defined convexities, concavities and inflections (intermediate points), segmented to isolate each feature type. Observers matched the segmented reference shapes to one of two subsequently presented whole-contour shapes (target or distractor) that were re-scaled and re-positioned. For very short segment lengths, performance was significantly higher for convexities than for concavities or intermediate points and for convexities remained constant with increasing segment length. For concavities and intermediate points, performance improved with increasing segment length, reaching convexity performance only for long segments. No significant differences between concavities and intermediates were found. These results show for the first time that closed curvilinear shapes are encoded using the positions of convexities, rather than concavities or intermediate regions. A shape-template model with no free parameters gave an excellent account of the data.

The visual system is exposed daily to a vast array of shapes and objects, both natural and artificial. Object recognition, categorization and classification, across a wide range of circumstances, is effortless^1^, fast^2^, and can be driven by either luminance or chromatic information^3^. Moreover object recognition is largely independent of naturally occurring variations such as in object position, scale, pose and illumination- the so-called invariance problem (reviewed by DiCarlo, Zoccolan, & Rust, 2012^4^). For example, in both the retinal image and on flat displays (drawings, photographs), shapes and objects are projected onto two dimensions, yet recognition is generally not impaired. The perceptual invariance of object recognition achieved by human vision continues to be a challenge for computer vision^5^. One important question is how the visual system represents the enormous variety of possible shapes.

There is a long history of theoretical and experimental investigations concerning the importance of specific shape features in shape recognition and object perception. Alhazen (965–1040 CE) suggested that points of convexities and concavities are crucial for the perception of shapes (Ibn al-Haytham, 1030/1989); convexities and concavities are the parts that curve outwards and inwards with respect to a reference point, such as the center of the object. Over 60 years ago, Attneave (1954) emphasized the significant role of regions of high curvature in shape perception and argued that:

*“…information is concentrated along contours at those points on a contour at which its direction changes most rapidly…”*

and he additionally proposed:

*“Common objects may be represented with great economy, and fairly striking fidelity, by copying the points at which their contours change direction maximally, and then connecting these points appropriately with a straightedge.”*

To underpin his hypothesis he took an image of a sleeping cat, extracted the points of maximum curvature (convexities and concavities) and connected them with straight lines, thus omitting intermediate shape information. Despite the absence of this intermediate contour information, the sleeping cat was still recognizable^6^. In another experiment (never fully published), subjects consistently selected convexities and concavities when asked to indicate the most salient points on a smoothly curved closed shape. These results have been confirmed in by Norman *et al*. (2001). In their study, subjects were presented with silhouettes of smoothly curved sweet potatoes and were tasked to position ten points along each shape’s outline which they considered to be the most informative. Results showed that subjects chose regions of high curvature of both signs, i.e., convexities and concavities^7^.

Other studies have drawn different conclusions with regards to the importance of convexities, concavities and intermediate points. Kennedy and Domander (1985) used fragmented familiar shapes (line drawings of, e.g., a box, a stove, a window, etc.) and showed that the areas of least curvature (regions halfway between concavities and convexities) are the most important for recognition, questioning Attneave’s earlier proposal. In contrast, Biederman (1987) removed zones of maximum curvature (vertices) or intermediate points (midsegments) from images of familiar objects and showed that removing the convex regions led to a larger impairment than removing either the concave or intermediate regions, suggesting a predominant role for convexity. Results on the detection of changes in shapes^8^ and visual search^9^,^10^ suggest that local concavities contain the important information. Other studies, for example those that measured the discriminability of targets placed on contours of varying curvature^11^, or positional discrimination^12^,^13^,^14^ or the detection of symmetry^15^ favor convexity as the critical feature. However, in a subsequent study, Bertamini, Helmy and Hulleman (2012) suggested that the previously found advantage for convexities in symmetry detection may be caused by the tendency to attend to convexities^16^. Yet other studies have reported no difference for convexity or concavity for tasks such as change detection^17^ or visual search^18^. For shape discrimination, Loffler *et al*. (2003) demonstrated that sensitivity is impaired when small gaps are introduced at points along the contour (convex curvature maxima, concave minima and points of zero-crossing). Occluding points of maximum convex curvature led to the highest threshold elevations, suggesting that radial frequency (RF) patterns are detected using points of local maximum curvature^19^. In another study, Bell *et al*. (2010) employed a compound adaptation paradigm^20^,^21^,^22^ in conjunction with a suprathreshold RF pattern with salient peaks and troughs. In this paradigm the contribution of individual parts of an RF pattern, namely contour maxima, minima and inflection points could be measured individually and from the results Bell *et al*. (2010) concluded that all parts of an RF contribute equally to shape coding.

Some investigators have also tried to illuminate the topic from a theoretical point of view. Formalized on the basis of Shannon’s mathematical definition of information^23^, Resnikoff (1985) provided a formal proof that information concentration is highest in regions of maximum curvature, independent of the sign of curvature. More recently, Feldman and Singh (2005) extended Resnikoff’s proposition and showed that for closed contours segments of negative curvature (i.e., concavities) contain greater information than corresponding regions of positive curvature (i.e., convexities). Note, however, that Feldman and Singh’s approach has been questioned^24^,^25^.

In summary, previous results on the role of specific shape features are inconclusive and appear to be dependent on both the task and the stimulus design (see also Bertamini & Wagemans, 2013^26^ for a recent review). Here, we introduce a novel class of closed shapes, which can be arbitrarily manipulated to create a wide range of shapes, with varying magnitudes of convex and concave curvatures. These stimuli can be mathematically characterized and used to describe complex natural and synthetic shapes. Our aim was to generate stimuli with natural object-like boundaries, but which were nevertheless unfamiliar (Stimulus examples are shown in Fig. 1). The use of abstract but natural, instead of familiar shapes minimizes the influence of cognitive information, such as memory access, and hence template matching^27^, categorization, eye-movement planning, object familiarity and context. In our task observers had to match a segmented reference shape, which showed only either convex, concave or intermediate segments, to one of two subsequently presented re-scaled and re-positioned whole-contour shapes (target or distractor). With such a task we were able to test the role of these features in shape recognition (Fig. 2).

## Results

Figure 3 shows the results for each compound shape in a different graph (A: 2–3–4; B: 1–2–5, C: 2–3–6, D: 3–5–8). Each plot shows percent correct as a function of segment length averaged across subjects (N = 3) for convexities (green), concavities (red) and intermediate segments (blue). The shaded regions illustrate ± SEM. The results are similar for the different compound shapes, but differ depending on the feature presented. For very short (dot-sized) segments lengths, performance is significantly better for convexities than for either concavities or intermediate points. Surprisingly, performance for convexities remains relatively constant as a function of segment length for all tested shapes. A one-factor repeated measures ANOVA with the segment lengths as factors revealed the following main effects: 2–3–4: F_6,12_ = 1.001, *p* = 0.47; 1–2–5: F_6,12_ = 10.1, *p* < 0.001; 2–3–6: F_6,12_ = 1.8, *p* = 0.179; 3–5–8: F_6,12_ = 1.92, *p* = 0.159. Subsequent post-hoc tests (Bonferroni corrected) showed that only for compound shape 1–2–5 was performance significantly different between segment length 1 and 7 (*p* < 0.01) and segment length 2 and 7 (*p* < 0.001). This analysis shows that performance is largely independent of segment length. The average percent correct responses across all segment lengths was: 2–3–4: 81.6% (SEM ± 4.0%); 1–2–5: 80.3% (SEM ± 2.2%); 2–3–6: 88.6% (SEM ± 4.0%) and 3–5–8: 82.6% (SEM ± 2.1%).

To determine whether performance for convexities was dependent on the type of compound shape, a two-way repeated measures ANOVA with factors *shapes* and *segment length* was conducted. There was no significant effect of shape (F_3,6_ = 2.87, *p* = 0.126) or segment length (F_6,12_ = 192.2, *p* = 0.133). Additionally no significant interaction was found (F_18,37_ = 0.62, *p* = 0.862). In summary, the ability to recognize the shape from convexities is independent of segment length and type of compound shape.

In contrast to convexities, performance for concavities and intermediate segments improves gradually from close-to-chance for the shortest segment lengths to that of convexities at the longest segment length. The results for the shortest, dot-sized segments for all tested shapes and shape features are given in Table 1.

As the Table shows, for the dot-sized segment lengths, performance is on average around chance level for concavities (58.1 ± 2.8%) and intermediates (57.7% ± 2.5%) but much higher for convexities (80.7 ± 1.9%). As Fig. 3 shows, although performance improves with segment length for concavities and intermediates, it only reaches convexity performance at the largest segment lengths. Results were analyzed with a three-way *ANOVA* with factors of *shape feature* (concavity & intermediate), *shapes* (2–3–4, 1–2–5, 2–3–6, 3–5–8) and *segment length* (7 different lengths). This analysis revealed no significant differences between concavities and intermediates (F_1,2_ = 0.251, *p* = 0.67) and *shapes* (F_3,6_ = 2.544, *p* = 0.152). However, performance increases significantly with increasing *segment length* (F_6,12_ = 42.47, *p* < 0.0001). No significant interactions were found between factors (all *p* > 0.05).

Bertamini *et al*. (2012) conducted a series of experiments on symmetry perception and suggested that an advantage for convexities in symmetry detection may be caused by the observers’ tendency to attend to convexities^16^. In order to investigate this possible explanation, an additional naïve participant was recruited and performed the same task for compound shape 1–2–5 with a presentation time of just 180 ms. The observer repeated 3 blocks for each feature (convexities, concavities and intermediate). The results were averaged across blocks and are presented in Fig. 4A, the shaded error regions representing standard deviations. The grey-shaded error regions in Fig. 4B show 95% confidence intervals of the results for compound shape 1–2–5 from the other three observers (Fig. 3B), with SB’s results superimposed. Results clearly show the same pattern of results: performance for convexities remains constant as a function of segment length, whereas performance increases with increasing segment length. Performance is independent of presentation time down to 180 ms. This demonstrates that attentional effects cannot explain the results.

In summary, performance for concavities and intermediate segments increases with increasing segment length. No significant differences exist between concavities and intermediates. Performance is also independent of shape type.

## Model

We propose a model with no free parameters in which the visual system creates a ‘shape primitive’ in the form of a polygon formed by combining the endpoints of the segments in the reference shape. The polygon constitutes a template shape that is compared to both the whole-contour target and distractor shapes. The decision rule is to select the shape, either target or distractor, most similar to the template. The model is illustrated in Fig. 5. The metric for comparing the template with the target and distractor shapes is based on the *turning angle function*, a function designed to compare two polygons^28^. This metric has the important property that it is invariant with respect to translation, rotation and change of scale. The metric employs a pie-wedge sector, whose origin is the center of the shape and whose sides intersect with the shape at two points along its circumference. The two points are connected by a straight line (solid green line in Fig. 6). The angles *θ*_*Tem*_, *θ*_*Tgt*_ and *θ*_*Dis*_, which refer to the angles subtended by the gradients for the template shape, target shape and distractor shape respectively, were calculated as follows:


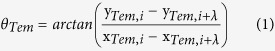



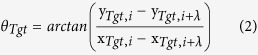



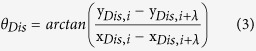


The central angle *λ* of the pie-wedge sector was set to 5°, which resulted in 72 values around the circumference of each shape (360°/5° = 72). Figure 6 exemplifies this procedure for the comparison between a reference and target shape. The 72 measurements were subsequently squared and summed to give an average difference measure (∆*θ*) between the template shape (*Tem*) and whole-contour target shape (*Tgt*) and distractor shape (*Dis*) (∆*θ*_*Tem vs. Tgt*_ & ∆*θ*_*Tem vs. Dis*_).


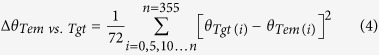



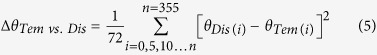


This procedure was simulated for 1500 randomly generated reference shapes and the corresponding target and distractor shapes, for each compound shape, i.e. RF combination (2–3–4; 1–2–5, 2–3–6, 3–5–8) and for each of the seven different segments lengths placed at convexities, concavities and intermediates. The Min Rule was applied; that is a smaller outcome translates to a closer similarity between the template and either distractor or target shape. This procedure was utilized for each segment length. The results from the simulations are percent correct of the model observer. The results were averaged across compound shapes and are illustrated by the solid lines in Fig. 7, whereas the solid circular data points show the psychophysical data averaged across subjects.

The shaded areas represent ± SEM of the data averaged across compound shapes. The goodness of fit between the model and the data was evaluated by calculating the coefficient of determination *R*^2^ for convexities (*R*^2^ = 0.89), concavities (*R*^2^ = 0.91) and intermediates (*R*^2^ = 0.92), demonstrating very good performance of the model. It is worth reiterating that the proposed model has no free parameters.

## Discussion

Our results show that for dot-sized segment lengths performance is significantly better for convexities than for either concavities or intermediate points. For convexities, performance remains constant as a function of segment length, for concavities and intermediate segments performance increases with increasing segment length. These results suggest that points of local curvature convexities are the critical features for recognizing the class of shapes tested here. This is in agreement with Biederman (1987), who demonstrated that the ability to recognize familiar shapes is significantly impaired when convex features (vertices) were removed. Our results add to the existing literature on convexities by showing that the predominant role of convexities in shape recognition also applies to non-familiar complex shapes. Furthermore, for the specific shapes used here, the ability to recognize the shape from convexities is independent of segment length or compound shape type and is scale-invariant.

Dot-sized points at the position of convex curvature maxima are sufficient to extract crucial information for the observer to match it to a whole-contour shape. It is a surprising and novel result that performance is independent of the complexity of the shape, as performance is similar for smoothly curved shapes possessing a low number of curvature maxima and minima (2–3–4) and shapes with many, salient curvatures (e.g. 3–5–8). Our results suggest that the information contained in between convexities is largely redundant. Performance for concavities and intermediate points on the other hand is around chance level for dot-sized segments, improves gradually with increasing segment length and reaches the same level as convexities for longer segment lengths. No significant differences were found between concavities and intermediates.

Independent of whether from a mathematical point of view more information is contained in curvature convexities or concavities, which remains a controversial issue^24^,^25^,^26^,^27^,^28^,^29^,^30^, our results are clear in that contour convexities are the most informative for shape recognition and that information contained in concavities and intermediate parts is largely redundant.

We speculate that the human visual system has developed a strategy that uses convex curvature maxima to encode whole shapes instead of using other features, i.e. concavities and intermediate points. Given that we found for our stimuli that only dot-sized points positioned at convexities were required to recognize a shape, we further propose a novel and simple model of whole-shape recognition in which the visual system creates polygon-like template shapes, by joining the endpoints of the convexities. This is analogous to a rubber band stretched around these points, i.e., forming a convex hull.

### Why are convexities special?

Any conceivable closed shape must contain the same number or more convexities than other shape features, such as concavities. Moreover, convexities always represent the outer boundary, i.e. the hull, of closed shapes. That is, if one connects convex curvature maxima in any closed shape, the resulting polygon envelops most of the shape and especially its concavities and intermediate regions. However, polygons created by connecting either concavities or intermediate points do not do this, as convexities tend to extend beyond the polygon’s confines.

### A sparse coding scheme for shape encoding

Many models of object recognition^31^,^32^,^33^,^34^ are based on the idea that the neural encoding of objects is achieved by a modular architecture along the ventral pathway^35^,^36^. This modular architecture involves increasing receptive field size, increasing invariance to position and scale and increasing stimulus complexity^37^,^38^. In addition it has been suggested that the vast amount of visual information that the brain receives is encoded by a relatively small number of active neurons out of a large population, an encoding strategy commonly referred to as ‘sparse coding’^39^,^40^,^41^. Sparse coding is believed to increase representational capacity and result in a reduction of metabolic demand^42^,^43^.

Carlson *et al*. (2011) showed that neurons in extrastriate area V4 are selectively responsive to stimuli with acute high curvatures. In addition, they analyzed images of familiar objects from the HEMERA Photo-object database (Hemera Technologies Inc.) with respect to the distribution of curvatures and showed that smooth low curvatures are much more common than acute curvatures (an order of magnitude). They suggest that the sparse coding of objects is reflected in the bias of V4 neurons towards less frequent object features, i.e., acute curvature maxima, and a more pronounced representation of uncommon, but salient (diagnostic) shape features, which contain most information about object identity. The authors suggest that sparse coding for uncommon, yet informative or diagnostic features could be a general encoding strategy of high-level visual processing^44^,^45^,^46^.

The superior and shape-independent performance for convex shape features observed here is supportive of Carlson *et al*.’s (2011) hypothesis that shapes might be encoded from specific salient shape features. Our experimental and modeling results suggest that convex curvatures in particular, rather than concavities and intermediate regions, are utilized to perform this recognition task. The information from shape features other than convexities seems to be less important for the recognition task employed here. However, these features might become more important for other types of stimuli or perceptual tasks. Otherwise, the visual system would not be able to distinguish shapes that only differ in concavities or intermediate regions.

The kind of segmented stimuli used here are reminiscent of partly occluded shapes. Previous studies on curve completion (interpolation) (e.g. Ben-Yosef & Ben-Shahar, 2012)^47^ have tested mathematically complex methods to predict the path of a segmented curve (tangent bundle). We present a method by which the simplest interpolation, a straight line connecting the endpoints of the segments, predicts the observed results.

## Methods

### Subjects

Three non-experienced observers who were also naïve as to the purpose of the study participated in this study. All had normal or corrected-to-normal visual acuity. Informed consent was obtained from each observer. All experiments were approved by the McGill University Ethics committee and were conducted in accordance with the original Declaration of Helsinki.

### Apparatus

The shapes were generated using MATLAB (MATLAB R 2013a, MathWorks) and presented on a gamma-corrected Iiyama Vision Master Pro 513 CRT monitor running with a resolution of 1024 × 768 pixels and a frame rate of 85 Hz (mean luminance 38 cd/m^2^), under the control of an Apple Mac Pro (3.33 GHz). Observers viewed the stimuli at a distance of 120 cm. At this viewing distance one pixel subtended 0.018˚ of visual angle. Experiments were performed in a dimly illuminated room. Routines from the Psychophysics Toolbox were employed to present the stimuli^48^,^49^.

### Stimuli

We aimed to create a novel class of stimuli with natural object-like boundaries. To this end we used compound radial frequency (RF) patterns. Combinations of different RF patterns have been employed previously to describe complex shapes, such as human heads^50^,^51^,^52^. The stimuli used here were composed of the sum of three different RF components, defined as follows:


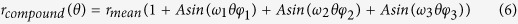


Where *r* (radius) and *θ* refer to the polar coordinates of the contour and *r*_*mean*_ the radius of the modulated circle, which was set to 100 Pixels (1.86˚ visual angle). The modulation amplitude *A* was set to 0.1. The variables *ω*_1_, *ω*_2_ and *ω*_3_ define the radial frequencies and *φ*_1_, *φ*_2_ and *φ*_2_ the phases (orientations) of each RF component, respectively. Four different RF combinations (*ω*_1,_*ω*_2,_*ω*_3_) were tested with frequencies of: 2–3–4, 1–2–5, 2–3–6 and 3–5–8. We refer to these as compound shapes. The highest radial frequency (*ω*) determines the maximum number of cycles in the compound shape. The phase (*φ*) of each RF component was randomly varied on each trial, such that observers never saw the same shape twice. Thus observers were presented with a wide range of shapes, varying from shapes with smooth curved boundaries (e.g., with RF components: 2–3–4) to shapes with very salient convexities and concavities (e.g., with RF components: 3–5–8). The shapes were black on a mid-grey background and had a width of 4 pixels (0.07˚ visual angle).

**Figure 1:**
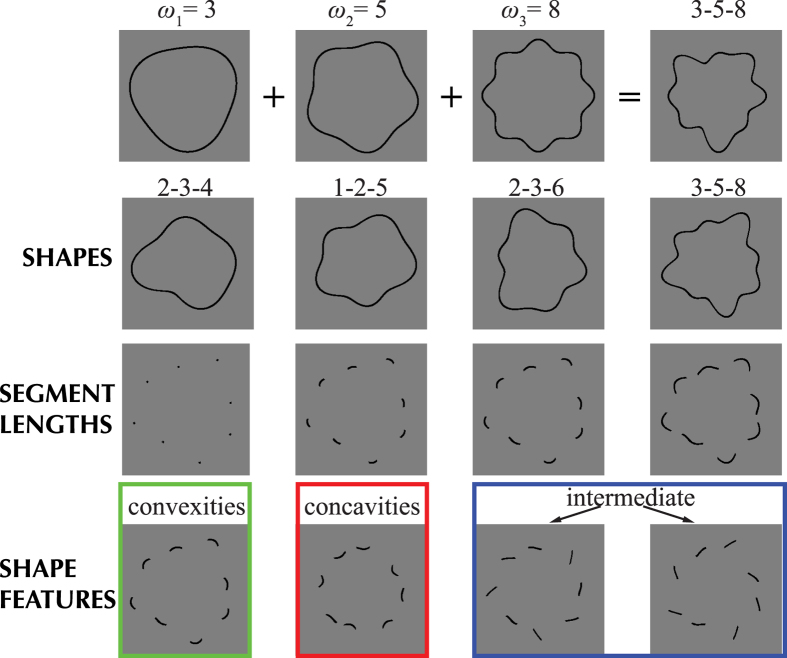
The top row shows the combination of three radial frequency (RF) patterns (RF3, RF5 & RF8) into a compound shape (defined by Eq. 1). The modulation amplitude *A* in all components was set to 0.1, whereas the phase *φ* (orientation) was randomly selected for each component. The second row shows examples of the four different shape combinations, or compound shapes that were tested (2–3–4, 1–2–5, 2–3–6 and 3–5–8). The third row shows different segment lengths, ranging from short (dot-sized) to longer segment lengths. The bottom row shows the segments centered on convexities, concavities and intermediate points.

In order to vary the segment length of the feature of interest, the maximum and minimum radii along the circumference of the shape were first determined to find the points of convexities and concavities, respectively. Intermediate points were defined as the angular midpoint between these convexities and concavities. Hence, the shapes contain twice as many intermediates as convexities and concavities. In order to allow a valid comparison, we presented only every second intermediate segment on a given trial. The position of these intermediate segments was randomly changed each trial (see Fig. 1, bottom row). An iterative method was used to add an equal amount of visible contour to both sides of the features of interest according to the specified segment length. Seven different segment lengths, ranging from dot-sized marks at the position of the contour feature to longer segments were employed for each type of compound shape (2–3–4, 1–2–5, 2–3–6, 3–5–8). The shorter segment lengths were equal for all compound shapes, whereas the longer and the maximum segment lengths were pre-determined individually for each compound shape, in order to avoid overlap of neighboring segments. The length of the contour was on average different for each type of compound shape and also varied somewhat for different presentations of the same compound shape. The segment lengths were defined as the percentage of the total contour length.

The noise-shapes (distractor) were composed of the same RF components as the segmented reference shapes, but with different pseudo-randomly determined phases. Example stimuli are shown in Fig. 1.

### Procedure

**Figure 2:**
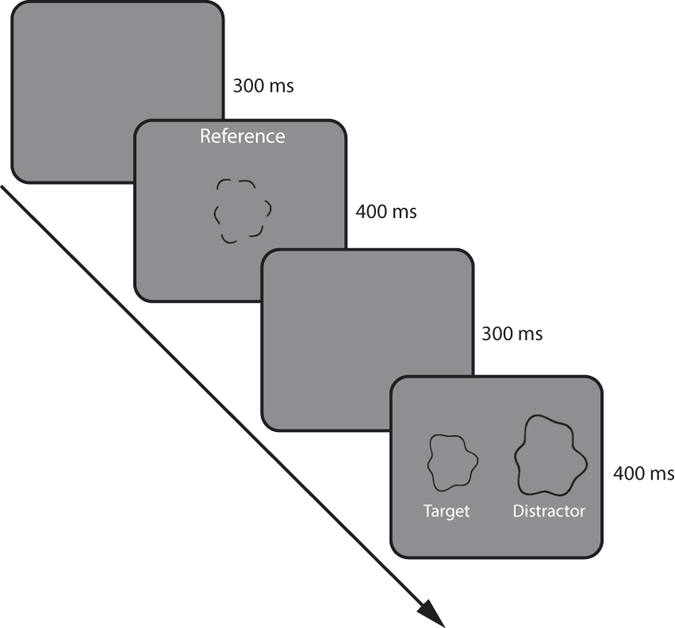
The task for the observers was to match a segmented reference shape to one of two whole-contour shapes, one a distractor- the other a target-shape.

**Figure 3:**
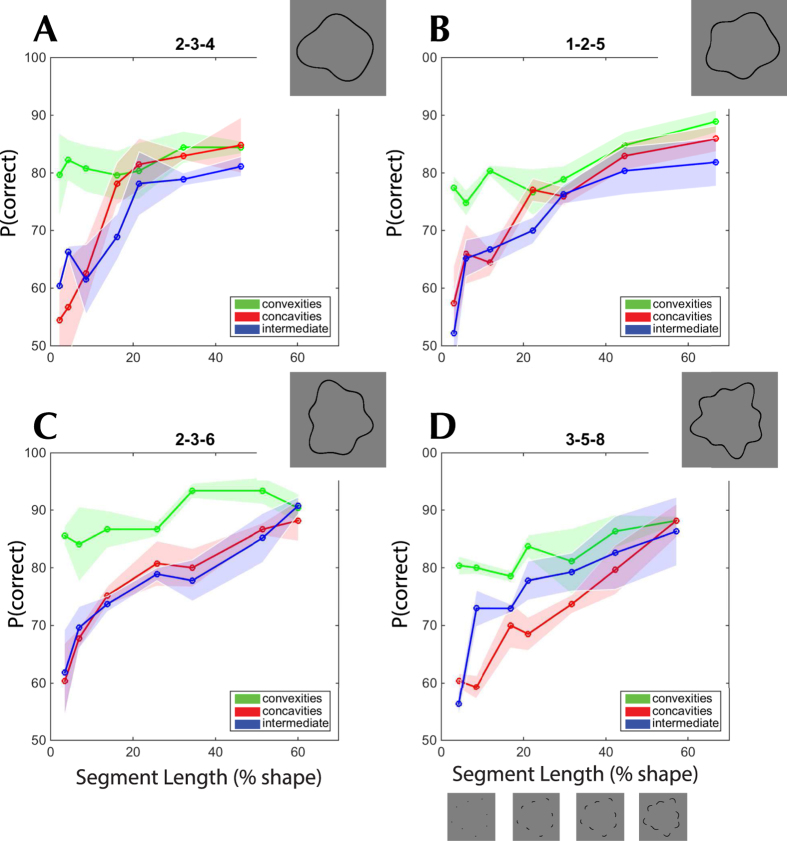
The graphs show percent correct responses as a function of segment length for four different shapes (A) 2–3–4; (B) 1–2–5; (C) 2–3–6; (D) 3–5–8) averaged across subjects (N = 3). The icons in the top right corner of each graph show stimulus examples. The icons along the abscissa of D illustrate different segment lengths. The green line shows mean results for convexities, the red line for concavities and the blue line for intermediate points. The shaded areas represent ± SEM.

**Figure 4:**
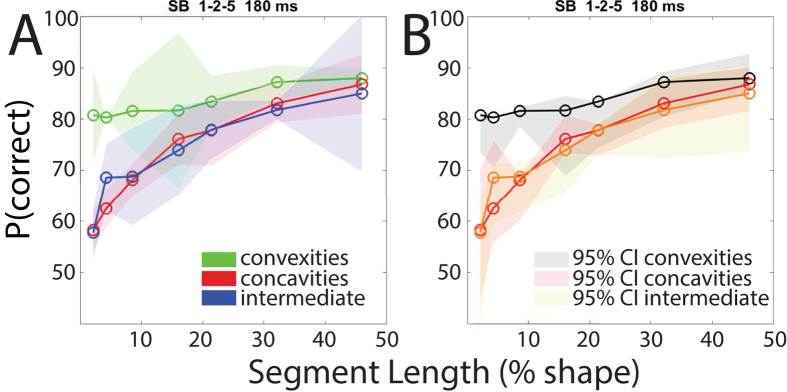
(**A**) The graph shows percent correct responses as a function of segment length for compound shape 1–2–5 for one additional naïve subject (SB), for a stimulus presentation time of 180 ms. The green line shows mean results for convexities, the red line concavities and the blue line for intermediate points. The shaded areas represent ± STDEV for this observer. (**B**) Figure 4B shows 95% confidence intervals (CI) for the data presented in Figure 3B (N = 3, presentation time 400 ms) with the data from the naïve subject (SB at 180 ms) superimposed.

**Figure 5:**
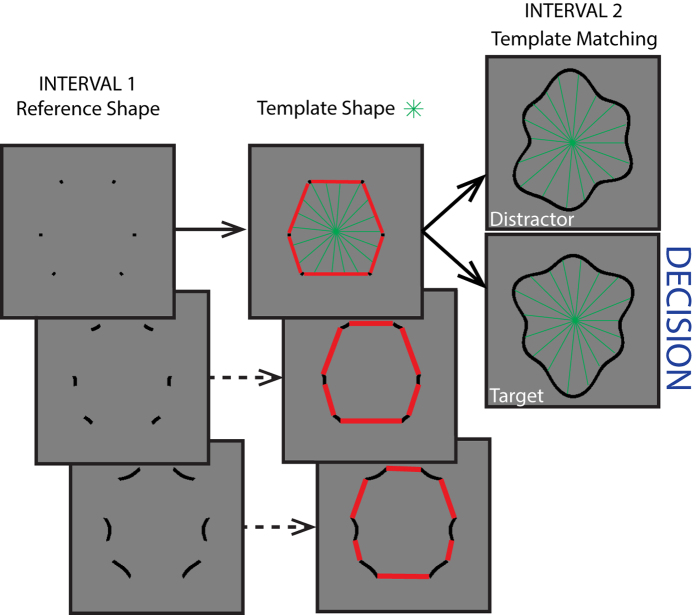
The model assumes that visual system generates a template shape by connecting the endpoints of the convex, concave (shown here) or intermediate segments in the reference shape with straight lines. The resulting template shape is then compared to the whole-contour target shape and distractor shape. The method used to compare the two shapes is described in the text and Figure 6. The decision rule is to select the shape (target or distractor) most similar to the reference shape template.

**Figure 6:**
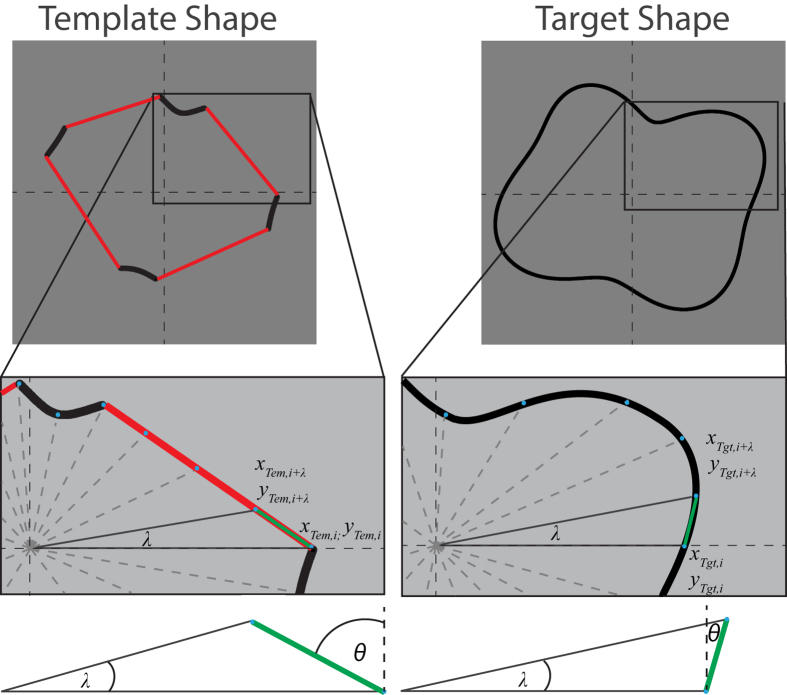
Comparison of two shapes, illustrated for a template shape (left) and target shape (right), shown for concavities of a specific segment length. See text for details.

**Figure 7:**
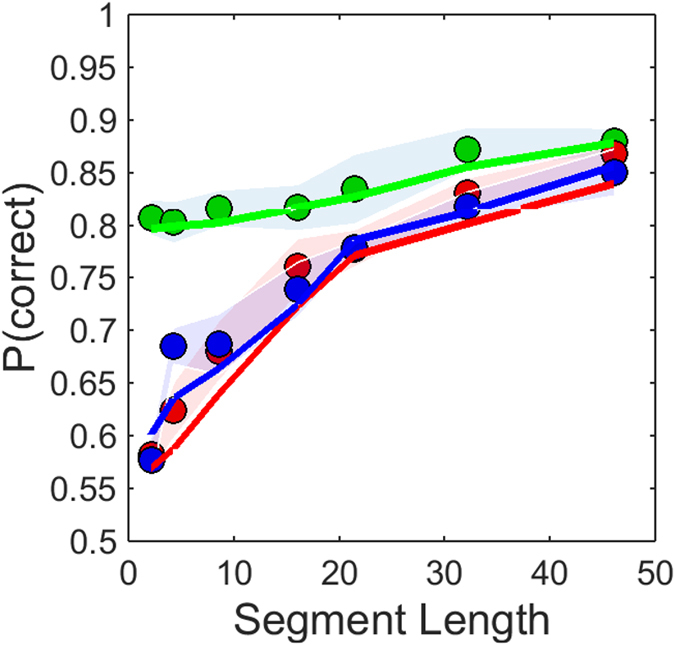
Data and model prediction. The solid circular dots represent the psychophysical data averaged across compound shapes (2–3–4; 1–2–5, 2–3–6, 3–5–8). The green dots show mean results for convexities, red dots for concavities and blue dots for intermediates. The shaded areas represent ± SEM. The solid lines illustrate the model performance for convexities (green), concavities (red) and intermediates (blue). See text for details.

Using a Match-to-Sample task, observers were asked to match a segmented reference shape to one of two whole-contour shapes; a distractor shape and a target-shape. The segmented reference shape was presented in the center of the screen. The distractor and target-shapes were presented simultaneously and their locations were randomly determined on a trial-by-trial basis (left or right side of the display). A vertical and a horizontal jitter (±60 pixels) was also applied. Furthermore, the target and distractor shapes were each scaled in size by a random amount in the range: −1/2 to +1/4 relative to the reference shape. This scaling minimized the possibility of observers simply judging the distance between shape features but was presumed not to impair performance significantly due to the scale-invariant nature of shape recognition (see Introduction). An experimental trial consisted of a 400 ms presentation of the segmented reference shape followed by a 300 ms blank interval (mid-grey blank screen), followed by a 400 ms presentation of the two whole-contour stimuli. A blank screen followed, during which observers were free to make their choice - choose the whole-contour shape that matched the segmented reference shape – and register their response by choosing left or right on a numeric keypad. The key press initiated the next trial. Auditory feedback was provided for incorrect responses. The experimental paradigm is illustrated in Fig. 2.

A blocked design was employed. Each combination of compound shape (2–3–4, 1–2–5, 2–3–6 & 3–5–8) and shape feature (convexities, concavities and intermediates) was tested in a separate block. The seven different segment lengths were each tested 30 times, resulting in a total number of 210 trials per block. Subjects typically repeated each block three times.

## Additional Information

**How to cite this article**: Schmidtmann, G. *et al*. Shape recognition: convexities, concavities and things in between. *Sci. Rep*. **5**, 17142; doi: 10.1038/srep17142 (2015).

## Figures and Tables

**Table 1:** Percent correct for the shortest (dot-sized) tested segment length for convexities, concavities and intermediate.

Shape	Convexities	Concavities	Intermediate
2–3–4	79.6% (SEM ± 7.3)	54.5% (SEM ± 8.9)	60.4% (SEM ± 0.9)
1–2–5	77.4% (SEM ± 2.1)	57.4% (SEM ± 6.6)	52.2% (SEM ± 6.8)
2–3–6	85.5% (SEM ± 1.7)	60.4% (SEM ± 6.6)	61.8% (SEM ± 7.5)
3–5–8	80.4% (SEM ± 1.6)	60.4% (SEM ± 1.3)	56.3% (SEM ± 2.1)
Mean	**80.7% (SEM ± 1.9)**	**58.1% (SEM ± 2.8)**	**57.7% (SEM ± 2.5)**
